# *Acinetobacter baumannii* Antibiotic Resistance Mechanisms

**DOI:** 10.3390/pathogens10030373

**Published:** 2021-03-19

**Authors:** Ioannis Kyriakidis, Eleni Vasileiou, Zoi Dorothea Pana, Athanasios Tragiannidis

**Affiliations:** 1 Pediatric and Adolescent Hematology-Oncology Unit, 2nd Department of Pediatrics, Faculty of Health Sciences, School of Medicine, Aristotle University of Thessaloniki, AHEPA Hospital, 54636 Thessaloniki, Greece; eleni.vasileiou@yahoo.com (E.V.); atragian@auth.gr (A.T.); 2 Laboratory of Cytogenetics and Molecular Genetics, Department of Biology, Faculty of Medicine, University of Thessaly, 41500 Larissa, Greece; 3 School of Medicine, European University, 1516 Nicosia, Cyprus; z.pana@euc.ac.cy

**Keywords:** *Acinetobacter baumannii*, antibiotic, resistance, antimicrobial, multidrug resistant, extensive drug resistant, pandrug resistant, next-generation sequencing, CRAB

## Abstract

*Acinetobacter baumannii* is a Gram-negative ESKAPE microorganism that poses a threat to public health by causing severe and invasive (mostly nosocomial) infections linked with high mortality rates. During the last years, this pathogen displayed multidrug resistance (MDR), mainly due to extensive antibiotic abuse and poor stewardship. MDR isolates are associated with medical history of long hospitalization stays, presence of catheters, and mechanical ventilation, while immunocompromised and severely ill hosts predispose to invasive infections. Next-generation sequencing techniques have revolutionized diagnosis of severe *A. baumannii* infections, contributing to timely diagnosis and personalized therapeutic regimens according to the identification of the respective resistance genes. The aim of this review is to describe in detail all current knowledge on the genetic background of *A. baumannii* resistance mechanisms in humans as regards beta-lactams (penicillins, cephalosporins, carbapenems, monobactams, and beta-lactamase inhibitors), aminoglycosides, tetracyclines, fluoroquinolones, macrolides, lincosamides, streptogramin antibiotics, polymyxins, and others (amphenicols, oxazolidinones, rifamycins, fosfomycin, diaminopyrimidines, sulfonamides, glycopeptide, and lipopeptide antibiotics). Mechanisms of antimicrobial resistance refer mainly to regulation of antibiotic transportation through bacterial membranes, alteration of the antibiotic target site, and enzymatic modifications resulting in antibiotic neutralization. Virulence factors that may affect antibiotic susceptibility profiles and confer drug resistance are also being discussed. Reports from cases of *A. baumannii* coinfection with SARS-CoV-2 during the COVID-19 pandemic in terms of resistance profiles and MDR genes have been investigated.

## 1. Introduction

Antimicrobial or antibiotic resistance (AMR) has emerged as a substantial and triggering phenomenon with increasing costs for healthcare systems worldwide. In recent years it has been related to significant morbidity, mortality, and increased cost due to both prolonged length of hospitalization and treatment. Although in the last decades, there are new antimicrobial agents in our armamentarium, resistance seems to be an expanding problem with geometric evolution. Data from multicenter studies in the last decades have demonstrated that both community-acquired and nosocomial AMR are increasing alongside the increased number of older patients with primary or secondary immunodeficiencies [1,2].

*Acinetobacter baumannii* belongs to the Moraxellaceae family and is a Gram-negative bacterium that predominantly causes nosocomial infections. These infections are diverse and may include hospital-acquired and ventilator-associated pneumonia (HAP, VAP), urinary tract infections, meningitis, bacteremia, and gastrointestinal and skin/wound infections [3,4,5,6,7]. 

*A. baumannii* is one of the ESKAPE organisms (*Enterococcus faecium*, *Staphylococcus aureus*, *Klebsiella pneumoniae*, *A. baumannii*, *Pseudomonas aeruginosa*, and *Enterobacter spp.*), that pose a global threat to human health and a therapeutic challenge due to emerging and constantly increasing resistance. Carbapenem resistant *A. baumannii* (CRAB) was ranked in 2018 by WHO as number one priority for antibiotic research and development. Carbapenem was chosen as a marker, because carbapenem resistance is usually associated with a broad range of co-resistance to other antibiotic classes [8]. 

The overall prevalence of multidrug-resistant strains in patients with *A. baumannii* HAP and VAP is estimated to be 79.9%, ranging from 56.5% in Argentina and 61.8% in Taiwan to 100% in Central America, Pakistan, Lebanon, Qatar, and Croatia, while its overall mortality can be as high as 56.2% [9]. The patterns of carbapenem resistance differ throughout Europe and also within the countries of the Arab League. Increased incidence of carbapenem resistant *A. baumannii* isolates has been observed in Northern and Eastern Europe as well as in the Levant countries of the Arab League (Iraq, Jordan, Lebanon, Palestinian territories, and Syria) [10,11]. 

Carbapenems were the preferred treatment of multidrug resistant (MDR) *A. baumannii* infections, but their prior use has led to increased incidence of carbapenem resistance during the last years [12]. Polymyxins are now widely used as the antibiotics of choice for MDR *A. baumannii* infections, although they were initially avoided due to systemic toxicities (nephrotoxicity and neurotoxicity) [13,14]. Extensive drug resistant (XDR) *A. baumannii* is called an isolate resistant to three or more classes of antimicrobials (penicillins and cephalosporins—including inhibitor combinations, fluoroquinolones, and aminoglycosides, resistant to carbapenems in most of cases), while pandrug resistant (PDR) *A. baumannii* is an XDR isolate resistant to polymyxins and tigecycline. Lately, extensively drug-resistant isolates have been led to the discovery of novel antimicrobials and the introduction of new treatment approaches [15,16].

**Figure 1:**
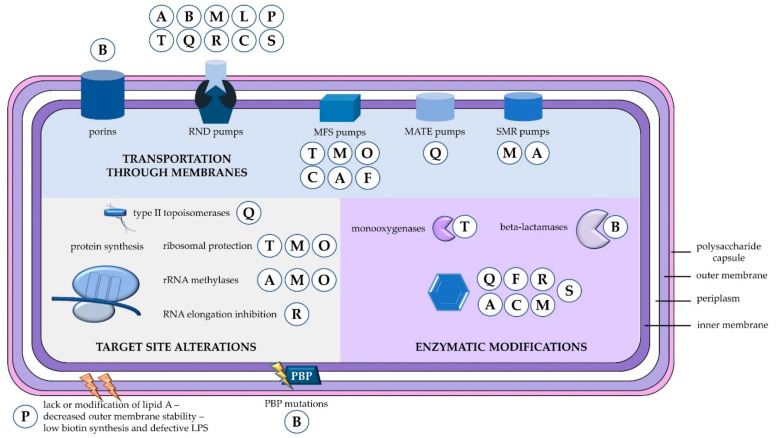
Mechanisms of antibiotic resistance in *A. baumannii*. Antibiotic resistance can be conferred through three main mechanisms, i.e., control of antibiotic transportation through membranes (reduction of porin permeability or increased efflux), modification of antibiotic targets, and enzymatic inactivation of the antibiotics. A = aminoglycosides; B = beta-lactams; C = chloramphenicol; F = fosfomycin; L = lincosamides; M = macrolides; MATE = multidrug and toxic compound extrusion; MFS = major facilitator superfamily; O = oxazolidinones; P = polymyxins; PBP = penicillin binding protein; Q = fluoroquinolones; R = rifamycins; RND = resistance-nodulation-division; S = diaminopyrimidines and sulfonamides; SMR = small multidrug resistance family; T = tetracyclines.

Mechanisms of antibiotic resistance can be categorized into three groups. First, resistance can be achieved by reducing membrane permeability or increasing efflux of the antibiotic and thus preventing access to the target. Second, bacteria can protect the antibiotic target through genetic mutation or post-translational modification, and last, antibiotics can be directly inactivated by hydrolysis or modification [17]. The mechanisms of *A. baumannii* antibiotic resistance based on this classification are summarized in Figure 1. One of the most important weapons in the armoury of *Acinetobacter* is its impressive genetic plasticity, facilitating rapid genetic mutations and rearrangements as well as integration of foreign determinants carried by mobile genetic elements. Of these, insertion sequences are considered one of the key forces shaping bacterial genomes and ultimately evolution [1,2]. Additionally, *A. baumannii* can form biofilms and thus prolong its survival on medical devices, such as ventilators in intensive care units (ICUs) [18]. However, the relationship between biofilm formation and antibiotic resistance still remains unclear [19,20]. In the present review, we report data on *A. baummannii* mechanisms of resistance to different classes of antibiotics and future perspectives for early identification of resistant genes.

## 2. Resistance to Beta-Lactams

Beta-lactams comprise of penicillins, cephalosporins, carbapenems, monobactams, and beta-lactamase inhibitors. Beta-lactam antibiotics are structurally similar to the d-Ala-d-Ala moiety of peptidoglycan, where penicillin-binding proteins bind, and thus they inhibit the transpeptidation, which is the last step in peptidoglycan synthesis [21]. *A. baumannii* is nowadays intrinsically resistant to penicillins and cephalosporins [22]. Resistance to beta-lactam antibiotics can be conferred through all the aforementioned mechanisms, which is inactivation by hydrolysis, increase of efflux, decrease of influx, and protection of the antibiotic target.

### 2.1. Beta-Lactamases

Beta-lactamases are enzymes that catalyze the hydrolysis of beta-lactam antibiotics and can be divided into four classes based on sequence motifs and differences in hydrolytic mechanism [23,24].

#### 2.1.1. Class A

Class A beta-lactamases mediate resistance to penicillin, cephalosporins, monobactams, and carbapenems. These lactamases may have narrow spectrum, or they can acquire extended spectrum antibiotic activity through point mutations. Narrow spectrum lactamases are active, mostly against penicillins, and can be inhibited by clavulanic acid [25], while extended spectrum beta-lactamases (ESBLs) can hydrolyze extended spectrum cephalosporins, like ceftazidime, ceftriaxone, cefotaxime, as well as aztreonam [26]. In addition, they are broadly distributed among Gram-negative bacteria with the help of plasmids and other mobile genetic elements [24]. Periodic surveillance of ESBL producing strains and detection of the respective genes (for example *blaTEM-92*, *blaSHV*, *blaGES-11*, *blaGES-14*, *blaPER-1*, *blaPER-7*, and *blaVEB-1*) can be of use in the clinical setting [27,28,29,30,31]. Other notable members of this class are the extended spectrum cefotaximases (CTX-M) and the *Klebsiella pneumoniae* carbapenemases (KPC) [32,33].

#### 2.1.2. Class B

Class B or metallo-beta-lactamases (MBLs) are encoded by mobile DNA (plasmids, integrons) and catalyze the hydrolysis of virtually all beta-lactamases (including carbapenems), but not monobactams, conferring multidrug-resistance. These enzymes require either zinc or another heavy metal for the catalysis and are further classified in three subclasses (B1, B2, and B3) based on sequence diversity and differences in the structure of their active sites. Moreover, four types of MBLs have been described in *A. baumannii*, namely IMP, VIM, NDM, and SIM [34]. The detection of MBL-producing organisms with the help of phenotypic methods, such as double disc synergy test, MBL E-test, and combined disk test, has been challenging [35,36]; however, molecular methods and in particular next generation sequencing are going to shed some light on their detection [37,38,39]. Phenotypic methods are not sensitive enough and thus do not detect all MBL producing strains [36]. With the help of PCR, *blaVIM-1* was detected in 14.3% of *A. baumannii* isolates characterized as MBL negative by E-test, highlighting the importance of introducing molecular methods into every-day practice in order to detect these hidden MBLs [40]. Recently, López et al. demonstrated that the expression of NDM lactamases does not compromise bacterial growth and is favored over other MBLs due to the lack of fitness cost leading to its worldwide dissemination among Gram-negative bacteria [41].

#### 2.1.3. Class C

Class C beta-lactamases are chromosomally encoded cephalosporinases (acinetobacter-derived cephalosporinase, ADC), inherent to all *A. baumannii*. Overexpression of these lactamases can be induced by the insertion of ISAba1 and ISAba125 sequences upstream of the encoding gene *blaADC* (formerly known as *blaAmpC*), which appear to be stronger promoters than the intrinsic promoter [42,43,44,45,46]. Insertions sequences (IS) are short transposable elements that are repeated multiple times throughout the genome, and thus are difficult to detect. An open-source bioinformatics pipeline (panISa) that utilizes whole genome sequencing (WGS) data as input has been recently developed in order to overcome this problem [47]. Several ADC variants have been described, many of which exhibit extended spectrum antibiotic resistance [48,49]. For example, ADC-30 provides resistance not only to cephalosporins, but also to carbapenems and sulbactam [50,51]. Finally, phosphoproteomic analysis revealed that dephosphorylation of ADC may lead to imipenem resistance in clinical isolates [52].

#### 2.1.4. Class D

Class D beta-lactamases, also called oxicillinases (OXA) or carbapenem-hydrolyzing class D β-lactamases (CHDLs), can inactivate all beta-lactams (mainly OXA-10 family) and comprise the main mechanism of carbapenem resistance. These enzymes are serine-dependent just like Class A and C beta-lactamases [53]. In addition, class D beta-lactamases usually cannot be inhibited by clavulanic acid, sulbactam, and tazobactam [54]. There are several *blaOXA* genes, including *blaOXA-51*, *blaOXA-23*, *blaOXA-24*, *blaOXA-58*, *blaOXA-143,* and *blaOXA-235*. The genes encoding these enzymes can be found on both the chromosome as well as the plasmids. Moreover, Wong et al. have recently confirmed that carbapenem resistance in clinical isolates of *A. baumannii* is mediated by over-expression of either OXA-23 or OXA-51 through insertion of ISAba1 in their promoter region [55]. With the help of molecular techniques (PCR, sequencing), reservoirs of carbapenemase-producing *A. baumannii* have been isolated from companion animals and pigeons, highlighting as a result the importance of global surveillance [56,57].

### 2.2. Outer Membrane Proteins

Antibiotic resistance due to beta-lactamases can be significantly enhanced when they collaborate with outer membrane proteins (OMPs). The low permeability outer membrane protein A (OmpA, 40 kDa) is the main non-specific porin in *A. baumannii* and has mainly a structural role [58,59]. It is speculated that OmpA takes part in the transport of antibiotics out of the periplasmatic space [60]. On the contrary, Iyer et al. demonstrated that OmpA selectively enables the uptake of small molecules like sulbactam, imipenem, and ETX2514 [61]. In mutant strains lacking OmpA (ΔompA and OmpA-like domain deletion), instability of the outer membrane and increased susceptibility to antibiotics (including penicillins and cephalosporins) has been observed [58,60,62]. Finally, Zhong et al. have recently shown that OmpA C-terminal domain is also responsible for anchoring beta-lactamases (Oxa23, GES-11) in the periplasmatic space, which may further explain the increased susceptibility in case of OmpA loss [63].

Mussi et al. have vigorously studied the carbapenem-associated outer membrane protein (CarO, 29 kDa) in carbapenem sensitive and resistant *A. baumannii* [64,65,66,67]. They have demonstrated that CarO serves as an uptake channel for L-ornithine and possibly carbapenems [66]. In carbapenem-resistant clinical isolates of *A. baumannii* insertion, elements can disrupt the expression of the chromosomal CarO gene and lead to complete loss of CarO [65,68,69]. In addition, CarO gene mutations can induce significant structural modifications in CarO and thus reduce outer membrane permeability and eventually cause drug resistance [70]. However, there are also studies that question the role of CarO in carbapenem resistance and suggest that other porin-mediated mechanisms might exist [71,72,73,74].

Srinivasan et al. studied the physiological functions of another OMP, AbuO, and demonstrated its role in multidrug resistance. AbuO is involved in the active efflux of multiple antibiotics, including ceftriaxone and meropenem [75]. Moreover, decreased expression of a different 33-36 kDa OMP is associated with carbapenem resistance [76,77]. Lastly, there have been contradicting studies regarding the involvement of the outer membrane carboxylate channel (Occ; formerly OprD) family in carbapenem resistance [78,79].

### 2.3. Efflux Pumps

Increased expression of efflux pumps contributes synergistically with beta-lactamases to antibiotic resistance [80]. Overexpression of AdeABC efflux pump is associated with *A. baumannii* carbapenem and cephalosporin resistance [81,82,83]. The AdeABC is a three-component efflux pump, member of the resistance–nodulation–division (RND) family. AdeB component expels antibiotics out of the cell, while AdeA is a membrane fusion protein and AdeC an outer membrane protein [84]. The substrates of AdeB can be diverse; they can range from hydrophilic to hydrophobic and can be either positively charged or neutral [83]. The expression of this efflux pump is regulated by the AdeRS two-component system. Point mutations in the *adeRS* operon can lead to increased expression of the pump and consequently to antibiotic resistance [85,86]. It has been shown that other efflux pumps AdeIJK and AbeM may also contribute to imipenem and cephalosporin resistance [87,88].

### 2.4. Penicillin-Binding Proteins

Penicillin-binding proteins (PBPs) are enzymes that catalyze the polymerization of peptidoglycan and are responsible for its insertion into the cell wall [89]. Beta-lactams bind to PBPs because they mimic their substrate. Inhibition of PBPs by beta-lactams then leads to an imbalance in cell wall metabolism and as a result to cell death [90].

The role of this resistance mechanism appears to be less significant but cannot be ignored. Although mutations in hot spot regions have been observed in PBP genes, the corresponding changes in amino acid sequences were not directly related to beta-lactam resistance [91]. However, Gehrlein et al. attributed imipenem resistance in a clone of *A. baumannii* strain No. 4852/88 to complex PBP alterations [92]. Moreover, Yun et al. reported increased expression of PBPs in a MDR *A. baumannii* strain (MIC of imipenem >128 µg/mL) as a response to imipenem exposure [93]. On the other hand, Fernández-Cuenca et al. observed reduced expression of a 73 kDa PBP [94] and Vashist et al. PBP alterations in carbapenem resistant *A. baumannii* [95]. Siroy et al. have also showed decreased expression of PBP1b in a clinical MDR isolate [96]. Finally, PBP3 mutants have been associated with resistance to meropenem, sulbactam, and cefiderocol [82,94,95,96].

In Table 1 are summarized the genes and the respective proteins that are involved in beta-lactam resistance retrieved from the pathogen detection microbial browser for identification of genetic and genomic elements (MicroBIGG-E), a database housed by National Center for Biotechnology Information (NCBI) [97].

## 3. Resistance to Aminoglycosides

According to MicroBIGG-E, resistance to aminoglycosides (AG) in *A. baumannii* can result through three distinct mechanisms (Table 2) [97]: aminoglycoside-modifying enzymes (AMEs) that weaken AG binding capacity, target site alteration by 16S rRNA methyltransferases, and limited AG uptake subsequent to loss of permeability or overactivity of efflux pumps. AMEs are further categorized as acetyl-, adenyl-, and phospho-transferases, depending on the site of AG modification (primarily by N-acetylation, O-nucleotidylation, and O-phosphorylation, respectively). *A. baumannii* displays intrinsic resistance to many antibiotics, while it seems to have acquired resistance to AGs in 19% to 31% of cases [98]. AGs are protein synthesis inhibitors that exert their action after crossing bacterial cell wall and by disturbing peptide elongation at the 30S ribosomal subunit. Genes conferring resistance can be transported by means of integrons, gene cassettes, transposons, and conjugated elements. Beyond the molecular level, and at cellular level, AG resistance genes can be transferred by means of mobilizable or conjugative plasmids, natural transformation, or transduction [99]. More specifically, AG resistance genes can be located in: (i) plasmid: *aac(3)-Ia, aac(3)-IVa, AAC(6’)-Ia family, aac(6’)-Ib, aac(6’)-Ib-cr, aac(6′)-Ih, aac(6’)-IIc, ant(2’’)-Ia, ant(3’’)-Ia, aadA1, aadA2, aadA5, aadA13, aadA16, aph(3’)-VIa, aph(3’)-VIb, aph(3’’)-Ib, aph(4)-Ia, aph(6)-Id,* and *armA*; (ii) integron: *aac(3)-Ia, AAC(6’)-Ia family, aac(6’)-Ib, aac(6’)-Ib’, aac(6’)-Ib3, aac(6’)-Ib-cr, aac(6’)-IIc, ant(2’’)-Ia, ant(3’’)-Ia, aadA1, aadA2, aadA5, aadA11, aadA13,* and *aadA16*; (iii) transposon: *aac(3)-Ia, AAC(6’)-Ia family, aac(6’)-Ib, aac(6’)-Ib-cr, ant(3’’)-Ia, aadA1, aadA5, aph(3’)-Ia, aph(3’)-IIa, aph(3’’)-Ib*, and *armA*; (iv) integrative conjugative element: *aph(6)-Id*, and *aph(3’’)-Ib*; (v) chromosome: *aac(2′)-Ib*, and *aph(3’’)-Ib*; or (vi) chromosomal genomic island: *aph(6)-Id* [100].

AME genes *aph(3’)-VIa*, *aph(3’)-VIb*, and *aph(3’)-VI* seem to be significantly more prevalent among amikacin and kanamycin resistant isolates, while isolates harboring *aac(6’)-Ian, aac(6’)-Ib, aac(6’)-Ib3, aac(6’)-I,* and *aac(6’)-Il* genes exhibit resistance against amikacin, kanamycin, and tobramycin [81,87]. Conversely, resistance to gentamycin should not be taken for granted in the presence of the above genes, while it is mainly mediated by 16S rRNA methylation (*armA* gene), 3-N- and 6’-N-acetylation of gentamycin, and by 2’’-O-nucleotidylation (*ant(2’’)-Ia* gene; correlated originally with resistance to kanamycin and tobramycin [97,103]. Unlike amikacin, gentamicin, and tobramycin that lack 3’-hydroxyl, groups usually retain activity in the presence of O-phospotransferases due to their inability to accept phosphate [104]. Resistance to AGs is increasing, and target site alteration seems to play a pivotal role with *armA* and *rmtB1* genes to be detected with plentiful of coexisting resistance genes [99].

Although AMEs remain the principal procedure by which *A. baumannii* evades elimination by AGs, efflux is also an emerging problem with AG use. Seven different gene products (Table 2) result in effective efflux of AGs, including pumps, permeases, periplasmic adaptors, and two component systems (TCSs) [105]. Of note, gentamicin and netilmicin are effectively cleared by AdeABC and AbeM pumps, but efflux is significantly weaker as regards more hydrophilic AGs, like amikacin and kanamycin [106]. Changes in membrane lipids and porin expression are only minor mechanisms of AG resistance and are still under investigation [99]. Recent case reports have indicated the usefulness of next-generation sequencing (NGS) in the prompt diagnosis and appropriate treatment of *A. baumannii* infections by early identification of resistance genes [107].

## 4. Resistance to Tetracyclines

Tetracycline antibiotics bind to the 30S ribosomal subunit and thereby inhibit protein synthesis by deterring the start of translation [108]. Resistance to tetracycline antibiotics is attributed to three main mechanisms: (i) efflux dependent on ATP, (ii) inactivation of tetracyclines by enzymes, and (iii) ribosomal protection proteins (RPPs) [109]. Table 3 describes resistance mechanisms that are unique for this antibiotic group.

Two types of efflux pumps that require energy are responsible for tetracycline resistance in *A. baumannii*. The resistance/nodulation/cell division (RND) family-type pumps are constitutive non-specific pumps originating from *adeA, adeB*, and *adeC* genes, which encode periplasmic adaptor subunits, permease subunits, and outer membrane pump elements, respectively [83]. RND pumps, and predominantly AdeABC, can effectively eliminate tetracyclines, while correspondingly, they mediate a substantial elevation of minimum inhibitory concentrations (MICs) for tigecyline, minocycline, and tetracycline [106]. RND pump AdeIJK seems to have a minor role in tetracycline resistance against *A. baumannii*, but it can act synergistically with other overexpressed efflux pumps (like AdeABC and AcrAB-TolC) and result in tigecycline resistance [115]. The second category refers to tetracycline major facilitator superfamily (MFS) efflux pumps: TetA and TetB [116]. TetA seems to lead efflux of tigecycline into the periplasm, and subsequently, RND pumps drive to elimination through the outer membrane [117].

TetM/TetW/TetO/TetS family tetracycline resistance RPPs abolish the inhibitory effect of tetracycline on protein synthesis by a non-covalent modification of the ribosomes [118]. Though rare, resistance to minocycline in *A. baumannii* has been attributed to ribosomal protection and *tetM* gene. The latter gene product mediates tetracycline release from its ribosomal binding site by a GTP-dependent mechanism, enabling the continuation of translation despite the presence of tetracycline [119].

Recent transcriptomic studies have shown that *A. baumannii* can swiftly become resistant to tigecycline [120]. Tigecycline has been a powerful tool against *A. baumannii* in the clinician’s armamentarium until recently, when various plasmid-mediated *tet(X)* gene variants emerged. Tet(X3), Tet(X4), and Tet(X5) are monooxygenases that can inactivate all tetracyclines even tigecycline and the recently authorized eravacycline and omadacycline [121,122].

## 5. Resistance to Fluoroquinolones

Quinolones are bactericidals with a broad spectrum that are characterized by a bicyclic core formation bearing resemblance to 4-quinolone. Quinolone antibiotics are mostly fluoroquinolones displaying efficacy against both Gram-negative and Gram-positive pathogens [123]. Regarding their mechanism of action, quinolone antibiotics interrupt DNA replication by averting bacterial DNA from loosening and being cloned. Quinolones exert their action by inhibiting the ligase activity of the type II topoisomerases, DNA gyrase, and topoisomerase IV, which normally induce supercoiling in collaboration with DNA nucleases. Disrupting ligase activity, bacteria remain with double-stranded DNA breaks and thus are led to cell death. Notably, quinolones primarily affect gyrase activity, while toxicity against topoisomerase IV is secondary (i.e., there is no proof of sole *parC* mutations without concomitant alterations in *gyrA*) [123]. Quinolone resistance occurs via three different mechanisms: (i) target mutations in gyrase and topoisomerase IV, which weaken the respective quinolone–enzyme interactions; (ii) plasmid-borne resistance mediated by Qnr proteins, the AMEs AAC(6′)-Ib-cr and AAC(6′)-Ib-cr5, and by plasmid-encoded efflux pumps; and (iii) chromosome-derived resistance resulted by either low expression of porins or overexpression of chromosome-encoded efflux pumps [123,124,125]. A recent review reported resistance of *A. baumannii* to fluoroquinolones between 50% and 73% of cases, while the respective resistance in developing countries during the last years displayed a marked increase reaching 75% to 97.7% [98,126]. Table 4 lists all recorded quinolone-specific resistance mechanisms for *A. baumannii.*

Quinolone resistance occurring secondary to overactive RND pumps has been long documented and is quite common [128]. Mutations in the TCS elements regarding both regulator (AdeR, primarily with polymorphisms D20N, A91V, A136V, and P116L) and sensor (AdeS, mainly due to polymorphisms G30D, A94V, G103D, G186V, and T153M) of AdeABC pump result in higher fluoroquinolone efflux. Mutations in *adeR* and *adeS* seem to trigger overexpression of the AdeABC efflux system (especially of the permease subunit *adeB* gene) and are associated with resistance to ciprofloxacin, norfloxacin, and ofloxacin [128]. Beyond AdeABC, efflux systems AdeIJK and AdeFGH are also utilized by *A. baumannii* to remove fluoroquinolones from the cell and result in high MICs. In addition, contribution of the multidrug and toxic compound extrusion (MATE) transporters AbeM and AbeS to *A. baumannii* resistance against quinolones is disputable. AbeM pump primarily affects the hydrophilic fluoroquinolones, norfloxacin and ciprofloxacin, rather than the hydrophobic ones (such as ofloxacin). In general, these non-RND efflux pumps confer low-level resistance to fluoroquinolones, even though some studies suggest otherwise [124,129].

Quinolone resistance-determining regions (QRDRs) refer mainly to alteration of target sites in gyrase (Ser83Leu, Gly81Asp, and Ser81Leu mutations preventing quinolones from binding its alpha-subunit) and topoisomerase IV (mutations Ser80Leu, Glu84Lys, and Gly78Cys, and Ser84Leu in its subunit C). Although a single point mutation in DNA gyrase is usually not enough for resistance to fluoroquinolones in *A. baumannii* (maybe only against levofloxacin; single *parC* mutations link with ciprofloxacin resistance), concurrent mutations within QRDR regions of the *gyrA* and *parC* genes are linked with significantly higher level of quinolone resistance [130,131]. Alterations in *gyrB* and *parE* genes are of minor significance [126].

Plasmid-mediated quinolone resistance (PMQR) has been recently identified as a clinical problem against *A. baumannii* infections, but generally confers still low-level (≤10-fold) resistance [123,132]. Qnr genes *qnrAI* (the first PMQR gene to be identified), *qnrB*, *qnrB19*, and *qnrS* encode members of the pentapeptide-repeat protein family (homologous to McbG and MfpA proteins), which originally inhibit gyrase action through competition with DNA for binding, leading to a decrease of DNA binding with topoisomerase and thus protecting enzyme–DNA complexes from the action of quinolones [97,132]. Due to the aforementioned DNA homology, these pentapeptide repeat-containing Qnr proteins can also interact with gyrase and topoisomerase IV and prevent quinolones from cleavage, leading eventually to aggregation and subsequent accumulation of double-stranded DNA breaks that would be, in this case, lethal to *A. baumannii* [133]. PMQR by AMEs AAC(6′)-Ib-cr and AAC(6′)-Ib-cr5 is attributed practically to mutant variants of 6’ aminoglycoside acetyltransferase-Ib gene (“-cr” implying ciprofloxacin resistance; W102R and D179Y mutations) that encode enzymes acetylating the C7 unsubstituted nitrogen in the piperazine ring of norfloxacin and ciprofloxacin [134]. The third category of plasmid-borne quinolone resistance refers to efflux pumps, but no such case has been documented for *A. baumannii* so far. QepA (quinolone efflux pumps A1 and A2; contributing to resistance to norfloxacin, enrofloxacin, levofloxacin, and ciprofloxacin among *rmtB*-positive *E. Coli*) and OqxAB (quinoxaline-di-N-oxide olaquindox) are the first described pumps of this category [127,133].

Chromosome-derived resistance to fluoroquinolones associates either with low influx rhythm by downregulated or dysfunctional porins, or with efflux pumps that are overactive. In fact, porins are virulence factors of Gram-negative bacteria that regulate cellular permeability through the outer membrane and are merely linked with carbapenem resistance (e.g., in Pseudomonas aeruginosa isolates with low OprD expression). Porin genes like *ompA*, *omp25*, *omp33*, *oprC*, *oprD*, *oprW*, *dcap*-like, and *carO* have been implicated in this resistance mechanism. Low relative expression of Omp25 and CarO porins seems to correlate with resistant *A. baumannii* strains, but resistance to quinolones has not been justified [111,135]. In the case of fluoroquinolones, chromosome-encoded efflux pumps (described in Table 2) and porin alterations alone do not seem to render significant clinical resistance to *A. baumannii* [123,129]. 

## 6. Resistance to Macrolides—Lincosamides—Streptogramin Antibiotics

Macrolide antibiotics are of little use in *A. baumannii* infections. Azithromycin, but no other macrolide, seems to inhibit mucin production, suggesting efficacy against ventilator-associated pneumonia. Azithromycin for nosocomial pneumonia due to *A. baumannii* for pediatric and adult patients in intensive care units (ICUs) is used in combination with other antibiotics. In the latter case, azithromycin’s mechanism of action differentiates from its established way of action (inhibiting translation and protein biosynthesis by attaching to the 50S subunit): suppression of ERK/JNK pathway phosphorylation (between extracellular signal-regulated kinases and c-Jun N-terminal kinases) and nuclear translocation of NF-κB (nuclear factor kappa-light-chain-enhancer of activated B cells) [136]. According to MicroBIGG-E database, resistance to macrolides in *A. baumannii* is attributed to (i) three 23S rRNA (adenine(2058)-N(6))-methyltransferases, encoded by *erm(B), erm(C)*, and *erm(F)* genes), (ii) ABC-F type ribosomal protection protein Msr(E) or *msr(E)*, and (iii) two macrolide 2’-phosphotransferases encoded by *mph(A)* and *mph(E)* [97]. The first two classes lead to resistance by modification of the target site, while the third class results in macrolide inactivation. In particular, Mph(A) and Mph(E) seem to confer resistance to erythromycin, clarithromycin, azithromycin, and oleandomycin, but only in the presence of specific regulatory proteins [137].

Additional literature search revealed several references to efflux pumps involved in macrolide resistance in *A. baumannii* infections. WGS confirmed recently the potential role of multidrug efflux MFS transporter AmvA (encoded by *amvA*) in erythromycin resistance [138,139]. Another WGS study identified transferable genetic elements in macrolide-resistant *A. baumannii* strains encoding the proton motive macrolide efflux MFS transporter and ribosomal protection protein mef(E)/mel (originally found in resistant streptococci) in an operon of MEGA-element (a DNA fragment named after the macrolide efflux genetic assembly). Unlike other mechanisms discussed above, MefE pumps do not result in higher MICs for lincosamides or streptogramins [140,141,142]. The tripartite MacA–MacB–TolC transporter in *A. baumannii* intersects inner and outer membranes so as to actively extrude macrolides. Main components of this transmembrane machine are the ATPase MacB, belonging to the ABC superfamily, and the membrane fusion protein MacA, conferring synergistically resistance to azithromycin and roxithromycin [143]. In addition, detection of the SMR family transporter AbeS (homologue of *E. Coli* EmrE) links with erythromycin resistance [144]. Another pump involved in macrolide-resistant *A. baumannii* is AdeABC efflux pump, regulated by base substitutions in the *adeRS* operon. Conversely, inactivation of the latter operon negatively affects biofilm formation and prompts decreased expression of AdeABC [145].

Lincosamides compounds lincomycin, clindamycin, and pirlimycin are of limited use as single antimicrobial agents in *A. baumannii* infections. Their mechanism of action resembles that of macrolides, by binding the ribosomal 50S subunit and specifically its 23S portion to a site corresponding to the peptidyl–transferase center. Consequently, plasmid- and transposon-derived 23S rRNA methyltransferases belonging to the *erm* family (erythromycin ribosome methylase) elicit combined resistance to macrolides, lincosamides, and streptogramins B (i.e., MLSB phenotype) [146]. Although resistance to lincosamides can occur through ribosomal modification, efflux, and drug inactivation, reports from *A. baumannii* strains are limited to efflux pumps. RND efflux pumps AdeABC, AdeFGH, and AdeIJK have been documented to significantly increase MICs of all lincosamides in several *A. baumannii* isolates [129,147,148,149]. Far from pumps, WGS revealed the link between ABC transporter Msr(E) and macrolide phosphotransferase mph(E) with MLSB phenotype [150,151,152].

## 7. Resistance to Polymyxins

As mentioned before, Gram-negative bacteria, such as *A. baumannii*, bear a semi-permeable outer membrane for insertion of essential elements and clearance of toxic compounds. Lipopolysaccharides (LPSs) reside on the outer surface and contain a negatively charged hydrophobic lipid A, which in turn interacts with the cationic non-ribosomal lipopeptides of polymyxins B and E (widely known as colistin). This interaction results in destabilization of the outer membrane, uptake of the polymyxins into the periplasm, and increased permeability by disrupting the integrity of both outer and inner membranes. Although the detailed mechanism of action is unknown, the hydrophobic tail of polymyxins seems to be crucial for the induction of membrane damage, suggesting a detergent-like mode of action [153].

Mechanisms of resistance to polymyxins in *A. baumannii* include (i) drug target alteration by LPS lipid A modification subsequent to mutations in the *pmrCAB* operon and *mcr* genes; (ii) mutations of *lpxA*, *lpxC*, and *lpxD* genes -encoding acyltransferases essential to lipid A biosynthesis and associated with lipid A deficiency; (iii) *lpsB*, *lptD*, and *vacJ* expression associated with permeability defects and osmotic resistance of the outer membrane, subsequently leading to markedly elevated MICs for polymyxins; (iv) insufficient concentration of cofactors constitutional for LPS formation, like biotin, which are essential for susceptibility to polymyxins; and (v) efflux pumps [153,154,155,156].

Operon *pmrCAB* includes *pmrC* gene encoding a phosphoethanolamine (PEA) transferase, along with *pmrA* and *pmrB* that encode the PmrA/PmrB TCS. Mutations in the latter TCS (and especially of *pmrA*) induce the overexpression of *pmrC*. PEA residue addition to the 4′-phosphate group site of lipid A hepta-acylated form is the most commonly reported modification in polymyxin-resistant *A. baumannii*, resulting in removal of negative charges and thus lowering the affinity of LPS to this drug class. The addition of 4-amino-L-arabinose and/or galactosamine to the phosphate groups of lipid A and to residues within the core oligosaccharide acts similarly. Likewise, PmrA/PmrB TCS can lead to polymyxin resistance by upregulating the transcription of NaxD deacetylase and therefore by modifying LPS lipid A by means of the deacetylated β-galactosamine. In addition, mutations in *miaA* gene (encoding the posttranscriptional regulator tRNA dimethylallyl diphosphate transferase) seem to act synergistically with mutations in *pmrA* towards producing polymyxin-resistant phenotypes [157,158]. In the same context, mutations in the *pmrC* paralogues *eptA-1* and *eptA-2* have been associated with increased expression and colistin resistance [153,159]. Other PEA transferases that affiliate with polymyxin-resistance are encoded by *mcr-1*, *mcr-4*, and *mcr-4.3* genes. Noteworthily, resistance emerging from MCR PEA transferases that was thought to be originally chromosomal (and therefore limiting its rapid distribution and dissemination) has been recently reported to be also carried by plasmids [160,161,162].

Biotin, which is essentially involved in fatty acid synthesis, contributes in a dose-dependent manner to LPS synthesis. Deletions in *lpsB*, a gene encoding a glycosyltransferase involved in biotin synthesis, have been associated with colistin resistance [163].

A recent WGS study on colistin-resistant isolates of *A. baumannii* identified linkage with *vacJ, zndP, pldA, ttg2C*, and *pheS* genes. VacJ is linked with the Vps-VacJ ABC transporter system, which is in charge of preserving LPS and phospholipids at the outer and inner leaflet of the outer membrane respectively. Correspondingly, PldA is designated to remove phospholipids in the outer leaflet of the outer membrane, while ZndP is a zinc-dependent peptidase A, upstream of PldA, that also has a pivotal role in the outer membrane processing. Ttg2C is an efflux ABC transporter upregulated in response to phenol exposure (an organic solvent that solubilizes the cell wall, acting equivalently with polymyxins). Moreover, mutations in *pheS* that encodes phenylalanine-tRNA ligase subunit alpha seem to play a role in colistin resistance [164]. Beyond the two aforementioned ABC transporters, and as stated before in text, overexpressed RND efflux pump MexAB-OprM correlates with colistin resistance [156,165].

## 8. Resistance to Others

### 8.1. Resistance to Amphenicols—Oxazolidinones

*A. baumannii* usually acquires resistance to amphenicols through five distinct mechanisms: (i) chloramphenicol O-acetyltransferases: type A-1, type A-2 CatII, type B-2 CatB11, type B-3 CatB3, and type B-3 CatB8 encoded by catA1, catA2, catB11, catB3, and catB8 genes, respectively; (ii) bifunctional type B-3 chloramphenicol O-acetyltransferase CatB8/aminoglycoside N-acetyltransferase AAC(6’)-Ib or catB8/aac(6’)-Ib’ found in *A. pittii*; (iii) chloramphenicol efflux MFS transporters: CmlA/FloR family (cml), CmlA family (cmlA), CmlA1, CmlA5, CmlA6, and CmlB1; (iv) chloramphenicol/florfenicol efflux MFS transporters FloR and FloR2; and (v) multidrug efflux pumps [97]. All the above mechanisms confer resistance to chloramphenicol; the second class confers combined resistance to both chloramphenicol and gentamicin, while the fourth class confers resistance to chloramphenicol and florfenicol [166]. Specifically, the presence of *cmlA5* induces resistance to chloramphenicol and thiamphenicol, while *craA* identification associates with loss of susceptibility to chloramphenicol, thiamphenicol, and florfenicol. Of note, single-component MFS family chloramphenicol/H+ antiporter CraA (a homologue of *E. Coli* MdfA and MdtM pumps) can act in synergy with RND AdeABC, AdeFGH, and AdeIJK transporters towards the amphenicol resistance phenotype, particularly when chloramphenicol acetyltransferases are absent [167]. In the same context, MDR *A. baumannii* strain 5075 (Ab5075) chloramphenicol resistance has been linked with efflux by an inner membrane permease encoded by *ABUW_0982* gene [168]. Moreover, a recent study identified 8 MDR *A. baumannii* isolates bearing *tet(X5)* in plasmids and two isolates with *tet(X6)* in their chromosomes that displayed resistance to tigecycline, tetracycline, ciprofloxacin, trimethoprim/sulfamethoxazole, and florfenicol [169].

Although decreased membrane permeability has been mainly attributed to carbapenem-resistance in *A. baumannii*, low expression of porin OmpA has been associated with chloramphenicol, aztreonam, and nalidixic acid resistance [60]. Another gene, *abrp*, encoding a C13 family peptidase has been linked with decreased cell membrane permeability, faster cell growth rate, and decreased susceptibility to chloramphenicol, tetracycline, minocycline, doxycycline, tigecycline, and fosfomycin [170]. Conversely, overexpression of global regulator SoxR led to increased susceptibility to chloramphenicol, by downregulating *abeS*, *abeM*, *adeJ*, and *adeG*, although *adeB* and *craA* expression remained intact [171]. Additionally, deletion of valine–glycine repeat G (*vgrG* gene), a component of the Type VI Secretion System (T6SS), which is considered as a crucial virulence factor of *A. baumannii*, led to reduced resistance to chloramphenicol [172].

Rather bizarre combinations of colistin with anti-Gram-positive antibiotics, like the oxazolidinone linezolid, have demonstrated efficacy and have been utilized in cases of MDR *A. baumannii* [173]. Protein biosynthesis inhibition is mediated by different mechanisms as regards oxazolidinones, chloramphenicol, and lincosamides, but their target sites on the 23S ribosomal RNA subunit may be overlapping. The most prevalent mechanisms for linezolid resistance are multidrug efflux pumps, base substitutions in domain V of the ribosomal RNA 23S (conferring resistance to oxazolidinones, macrolides, lincosamides, streptogramins, phenicols, pleuromutilins, and glycopeptide antibiotics), and/or the presence of a transmissible Cfr(B) 23S ribosomal RNA methyltransferase (conferring resistance to oxazolidinones, streptogramins, phenicols, and lincosamides). Interestingly, plenty of microorganisms carry multiple gene copies that encode the 23S rRNA subunit, while the observed linezolid resistance corresponds to the ratio of wild type to mutant 23S rRNA [156,174]. According to the Comprehensive Antibiotic Resistance Database (CARD), resistance to linezolid can also occur by mutations in the 50S ribosomal subunit P-site (which attaches the peptidyl-tRNA to the developing polypeptide chain), by the MFS antibiotic efflux pump LmrS (capable of extruding oxazolidinones, macrolides, phenicols, aminoglycosides, and diaminopyrimidine antibiotics), ClcD (a cfr-like 23S ribosomal RNA methyltransferase linked with resistance to oxazolidinones, lincosamides, streptogramins, phenicols, and pleuromutilins), and ABC-F ATP-binding cassette ribosomal protection protein encoded by poxtA (conferring resistance to oxazolidinones, tetracyclines, macrolide antibiotic, lincosamides, streptogramins, phenicols, and pleuromutilins) [156].

### 8.2. Resistance to Glycopeptide and Lipopetide Antibiotics

Zeocin is a formulation of phleomycin D1, a glycopeptide antibiotic of the bleomycin family. Resistance to zeocin is linked with phleomycin/bleomycin binding protein Ble-Sh (*ble-Sh* gene), while resistance to bleomycin is usually conferred by the binding protein Ble-MBL (metallo-beta-lactamase-associated *ble* gene) [175,176,177].

The outer membrane of Gram-negative bacteria is impenetrable to large glycopeptide molecules, and therefore *A. baumannii* displays intrinsic resistance to vancomycin. However, VanD-type vancomycin resistance histidine kinase VanS (*vanS-D* gene) has been reported in *A. baumannii* infection [97]. Contrariwise, colistin combination with glycopeptides (vancomycin, teicoplanin, and telavancin) and lipopeptides (like daptomycin) has been successfully employed in MDR *A. baumannii* infections [178]. Studies on glycopeptide- and lipopeptide-resistant *A. baumannii* isolates are lacking.

### 8.3. Resistance to Rifamycins

Resistance to rifampin (also known as rifabicin) in *A. baumannii* infections has been linked with mutations in the *rpoB* gene, which encodes rifamycin sensitive beta-subunit of RNA polymerase and averts RNA elongation just after adding the first nucleotides. Beyond rifampin, RpoB associates with resistance to all rifamycins (rifabutin, rifaximin, and rifapentine) [179]. Enzymatic modification by rifampin ADP-ribosyltransferases Arr, Arr-2, Arr-3, and Arr-4 impels to inactivation of rifamycins’ 23-OH position using NAD+ [97,180]. As mentioned above in text, RND multidrug efflux pumps AdeIJK and AcrAB-TolC are also potential mechanisms of rifamycin-resistant isolates [156]. Conversely, *fhuE* gene encodes an outer membrane protein transporter that is involved with iron acquisition and is upregulated in low iron conditions and seems to associate with increased susceptibility to rifabutin (200-fold more potent than rifampin). The previous finding was confirmed by successful in vivo treatment of extreme drug resistant *A. baumannii* with rifabutin/colistin combination [181].

### 8.4. Resistance to Fosfomycin

Intrinsic fosfomycin resistance in *A. baumannii* occurs predominantly as a result of two mechanisms: (i) fosfomycin efflux MFS transporter AbaF encoded (*abaF* gene); (ii) fosfomycin resistance glutathione transferases FosLL, FosA3, and FosA3/FosA4 family (encoded by *fos*, *fosA3*, and *fosA*, respectively), and FosB1/FosB3 family fosfomycin resistance bacillithiol transferase (encoded by *fosB*) [97,182]. Disruption of *abaF* has displayed an increase in fosfomycin susceptibility and a decrease in biofilm formation and virulence, suggesting a major role for this pump [183]. Of note, FosA impels the binding of glutathione to Fosfomycin C1 and thereby renders it inactive, while FosB opens enzymatically the epoxide rind of Fosfomycin after employing either bacillithiol or L-cysteine [156].

Fosfomycin sensitive pyruvyl transferase MurA has been recently proposed as a potential drug target for MDR *A. baumannii*. This transferase, whih has no counterparts in eukaryotes, catalyzes the pathway of peptidoglycan biosynthesis and is indispensable for cell integrity, while it is highly sensitive to inhibition from fosfomycin [184].

### 8.5. Resistance to Diaminopyrimidines—Sulfonamides

Antifolate antibiotics exert their action by inhibiting purine metabolism, and thereby DNA and RNA synthesis. Trimethoprim is a dihydrofolate reductase (DHFR) inhibitor (blocking tetrahydrofolic acid formation by dihydrofolic acid, an essential step of folate biosynthesis, which in turn is involved in the generation of many nucleotides and amino acids), while sulfonamides are known dihydropteroate synthase (DHPS) inhibitors (halting the conversion of para-aminobenzoate or PABA to dihydropteroate; including sulfamethoxazole, commonly used in combination with trimethoprim, and sulfonamides) [185]. Resistance against diaminopyrimidines in *A. baumannii* infections is mainly conferred by trimethoprim-resistant dihydrofolate reductases DfrA1, DfrA5, DfrA7, DfrA10, DfrA12, DfrA14, DfrA16, DfrA17, DfrA19, DfrA20, DfrA27, and DfrB1 (encoded by *dfrA1*, *dfrA5*, *dfrA7*, *dfrA10*, *dfrA12*, *dfrA14*, *dfrA16*, *dfrA17*, *dfrA19*, *dfrA20*, *dfrA27*, and *dfrB1*, respectively) [97]. A recent study of *A. baumannii* in turkey and chicken raw meat revealed that prevalence of *dfrA* was as high as 71% [186]. The latter antibiotic resistance gene seems to be a typical part of cassettes belonging to class I integron [187]. RND efflux pump MexAB-OprM consists of MexA (the membrane fusion protein), MexB (the carrier located in the inner membrane), and OprM (the transporter positioned in the outer membrane). MexAB-OprM associates with lack of susceptibility to most antibiotics, including trimethoprim and even colistin, when expressed in high levels [156,165]. As mentioned above in text, efflux pumps AdeIJK and LmrS confer resistance to trimethoprim [148]. Another pump, the RND plasmid-borne OqxAB, links with resistance to trimethoprim, quinolones, tetracyclines, glycylcycline, and nitrofurans [188,189].

In general, *A. baumannii* is intrinsically resistant to sulfonamides in approximately 71.3% of isolates [98]. Resistance, in these cases, is being materialized through two main mechanisms: (i) sulfonamide-resistant dihydropteroate synthases encoded by *sul1* and *sul2*, and (ii) RND efflux pump MexAB-OprM with or without MexR mutations [156,190].

## 9. SARS-CoV-2 and Resistant *A. baumannii* Coinfections

During the COVID-19 pandemic, coinfection with *A. baumannii* secondary to SARS-CoV-2 infections has been reported multiple times in literature. An isolate with OXA-23 has been responsible for an outbreak in COVID-19 ICUs of a tertiary Japanese hospital [191]. Incidence of secondary infections (mainly lower respiratory tract infections) attributed to *A. baumannii* has been reported to be as high as 1% of hospitalized COVID-19 patients in an Italian hospital [192]. The same incidence (1%) has been reported by an early study in hospitalized patients from Wuhan, China [193]. A concurrent study from Wuhan reported *A. baumannii* coinfection in one out of 69 hospitalized patients (1.4%) with COVID-19 [194]. In addition, a recent study from a French ICU calculated coinfection with *A. baumannii* at 1.1% (1 out of 92; susceptible to third generation cephalosporins) in severe SARS-CoV-2 pneumonia patients [195]. Higher incidence of MDR *A. baumannii* coinfection has been documented in a study from Egypt (2.7%; seven out of 260 COVID-19 hospitalized patients; susceptible only to tigecycline and fluoroquinolones; bearing resistance genes NDM-1, TEM, and CTX-M) [196]. A recent study in hospitalized COVID-19 patients from Spain showed that coinfection with *A. baumannii* was apparent in 2.4% of hospitalized patients (17 out of 712; 16 out of these 17 patients in ICU) and it has been the strongest determinant of mortality with OR 9.329 (95% CI: 2.289 to 38.02; *p* = 0.002; higher than sex, bacteriemia, and number of comorbidities) [197]. A study from an Iranian ICU reported coinfection with MDR *A. baumannii* in 17 out of 19 COVID-19 patients (89.5%; no MBL-producing strain; 52% resistance rate to colistin), while all these patients died [198]. *A. baumannii* was detected in 20% of samples acquired from COVID-19 patients in an ICU from Beijing, China. All of these cases were identified during late ICU admission [199]. Of interest, a recent study from a Mexican ICU assigned to COVID-19 patients revealed clonal dispersion of MDR AdeABCRS+ *A. baumannii* that was only susceptible to gentamicin, nitrofurans, and phenicols [200]. Steroids and long ICU stays, but not MDR infections, have been associated with higher mortality in an Italian retrospective analysis of 32 COVID-19 ICU patients. In the latter cohort, four patients have been coinfected with MDR *A. baumannii* (12.5%) [201]. CRAB outbreaks in COVID-19 patients seem to be effectively treated with a pre-optimized two-phage cocktail [202]. This finding is important, because OXA-23 CRAB infections have become a serious threat against SARS-CoV-2 infected patients in ICUs [203].

## 10. Conclusions

As justified above in text, *A. baumannii* may acquire antibiotic resistance through several distinct mechanisms: by altering the antibiotic target site, by controlling the passage of antibiotics through its membranes, and by enzymatic modification of antibiotics, rendering them neutralized. Secondary to innate mechanisms of antibiotic resistance that are de facto conferred by genes, *A. baumannii* may facilitate antibiotic resistance through various mechanisms linked with its virulence: outer membrane proteins (like porins), cell envelope factors (like LPS and the capsule around its bacterial surface), specific enzymes (like phospholipases C and D, and glycan-specific adamalysin-like protease CpaA), quorum sensing, and biofilm formation (BfmRS TCS regulating Csu pili, Csu expression regulated by the GacSA TCS, biofilm-associated proteins BapAb, synthesis of the exopolysaccharide poly-β-1,6-N-acetylglucosamine PNAG, acyl-homoserine lactones through AbaR receptor, and AbaI autoinducer synthase), by attaining twitching motility via type IV pili, micronutrient acquisition systems (like siderophores and iron transporters FecA and FecI, ZnuABC transporter, and ZigA GTPase incorporating a zinc-scavenging system, resistance-associated macrophage protein NRAMP for manganese transportation), type II (with CpaA and lipases LipA and LipH as effectors), and type VI protein secretion systems [1].

Utilization of next-generation sequencing techniques has helped clinicians to decipher the molecular mechanisms of antibiotic resistance in PDR isolates of *A. baumannii*, and WGS has emerged as a powerful tool in the clinician’s armamentarium. In the future, application of WGS and other NGS techniques at diagnosis could provide useful insights on the microbiologic behavior and virulence of each case of critical *A. baumannii* infection. Apart from timely diagnosis, detailed mapping of the genetic background of each isolate could provide useful information as regards antibiotic treatment (in terms of precision medicine) and identification of novel therapeutic targets [102,107,204].

Of special note is the contribution of molecular methods, such as PCR and NGS, to the prompt identification of MDR *A. baumannii* epidemics. Specifically, the detection of integrase genes (*intI1, intI2*) is significantly associated with the epidemic character of *A. baumannii* as well as antibiotic resistance to multiple antimicrobials [205,206,207]. In the Netherlands, integrons are found in 43% of MDR and 85.7% of XDR *A. baumannii* strains [208], while in China and Iran, the prevalence of integrase genes in total *A. baumannii* isolates is higher and reaches 69.6% and 94.3%, respectively [209,210]. This correlation of integrase gene presence and antibiotic resistance highlights the importance of regular surveillance in order to prevent outbreaks of hospital acquired MDR *A. baumannii* infections.

Except from specific clinical scenarios, MDR isolates are usually treated successfully with synergistic therapeutic combinations with either beta-lactamase inhibitor sulbactam or colistin, while last-line schemes involve combination of colistin with rifampin or polymyxin B with tigecycline. In the first case, we aim for inhibiting bacterial DNA-dependent RNA polymerase along with disruption of the bacterial membranes, while in the latter case, we aim for inhibiting bacterial protein biosynthesis (by attaching to the 30S rRNA subunit) in combination with loss of the bacterial membranes’ integrity. Of course, tigecycline remains the first choice for MDR strains isolated in the setting of the ICU, while phages (like Bφ-C62) are an alternative against increased antibiotic resistance in MDR isolates [98]. Clinical experience has confirmed efficacy of trimethoprim/sulfamethoxazole against carbapenem-resistant isolates, while isolates resistant to both carbapenems and sulbactam are preferred to be treated with either minocycline or doxycycline [98,211]. XDR and PDR strains remain a serious clinical problem and identification of resistance mechanisms of this staggering microbe will elucidate pathogenetic mechanisms and propose new therapeutic targets and agents.

## Figures and Tables

**Table 1:** Mechanisms of *A. baumannii* resistance to beta-lactams.

Mechanism of Resistance	Element Name	Resistance		Element Symbol (Gene)	Protein Products
Class A beta lactamases	class A broad-spectrum beta-lactamase TEM-1		extended-spectrum	*blaTEM-1*	WP_000027057.1 and others
	class A extended-spectrum beta-lactamase SHV-5			*blaSHV-5*	WP_011117369.1 and others
	class A extended-spectrum beta-lactamase SHV-12			*blaSHV-12*	WP_002904004.1 and others
	carbapenem-hydrolyzing class A beta-lactamase GES-5			*blaGES-5*	WP_012658785.1
	class A extended-spectrum beta-lactamase GES-11			*blaGES-11*	WP_001211000.1 and others
	class A beta-lactamase GES-12			*blaGES-12*	WP_063860500.1 and others
	inhibitor-resistant class A extended-spectrum beta-lactamase PER-1			*blaPER-1*	WP_001100753.1 and others
	class A extended-spectrum beta-lactamase PER-7			*blaPER-7*	WP_032495440.1 and others
	class A extended-spectrum beta-lactamase VEB-1			*blaVEB-1*	WP_000706731.1 and others
	class A extended-spectrum beta-lactamase CTX-M-15			*blaCTX-M-15*	WP_000239590.1 and others
	class A extended-spectrum beta-lactamase CTX-M-55			*blaCTX-M-55*	WP_015387340.1
	class A extended-spectrum beta-lactamase CTX-M-115			*blaCTX-M-115*	WP_035895532.1
	carbapenem-hydrolyzing class A beta-lactamase KPC-2			*blaKPC-2*	WP_004199234.1 and others
Class B metallo-beta-lactamases	subclass B1 metallo-beta-lactamase NDM-1		all (except monobactams)	*blaNDM-1*	WP_004201164.1 and others
	subclass B1 metallo-beta-lactamase IMP-1			*blaIMP-1*	WP_003159548.1
	subclass B1 metallo-beta-lactamase IMP-4			*blaIMP-4*	WP_015060105.1
	subclass B1 metallo-beta-lactamase IMP-14			*blaIMP-14*	WP_039819893.1
	subclass B1 metallo-beta-lactamase IMP-16			*blaIMP-16*	WP_063860576.1
Class C beta-lactamases	class C extended-spectrum beta-lactamase ADC-11		extended-spectrum	*blaADC-11*	WP_001211205.1 and others
	class C beta-lactamase ADC-25			*blaADC-25*	WP_001211217.1 and others
	class C extended-spectrum beta-lactamase ADC-26			*blaADC-26*	WP_001211238.1 and others
	class C extended-spectrum beta-lactamase ADC-30			*blaADC-30*	WP_001211218.1 and others
	cefepime-hydrolyzing class C extended-spectrum beta-lactamase ADC-33			*blaADC-33*	WP_001211220.1 and others
	class C extended-spectrum beta-lactamase ADC-52			*blaADC-52*	WP_001211232.1 and others
	cefepime-hydrolyzing class C extended-spectrum beta-lactamase ADC-56			*blaADC-56*	WP_031973850.1 and others
	class C extended-spectrum beta-lactamase ADC-73			*blaADC-73*	WP_001211219.1 and others
	class C extended-spectrum beta-lactamase ADC-74			*blaADC-74*	WP_001211203.1 and others
	class C extended-spectrum beta-lactamase ADC-76			*blaADC-76*	WP_001211237.1 and others
	class C extended-spectrum beta-lactamase ADC-79			*blaADC-79*	WP_001159760.1 and others
	class C extended-spectrum beta-lactamase ADC-80			*blaADC-80*	WP_029424536.1 and others
	class C extended-spectrum beta-lactamase ADC-82			*blaADC-82*	WP_001211216.1 and others
	class C beta-lactamase ADC-152		CEPH	*blaADC-152*	WP_001211233.1 and others
	class C beta-lactamase ADC-156			*blaADC-156*	WP_024436624.1 and others
	class C beta-lactamase ADC-162			*blaADC-162*	WP_031980335.1 and others
	class C beta-lactamase ADC-176			*blaADC-176*	WP_001159761.1 and others
	class C beta-lactamase ADC-182			*blaADC-182*	WP_057691114.1 and others
	class C beta-lactamase ADC-212			*blaADC-212*	WP_031975357.1 and others
	class C beta-lactamase ADC-222			*blaADC-222*	WP_031960999.1 and others
Class D beta-lactamases (oxicillinases)	carbapenem-hydrolyzing class D beta-lactamase OXA-23		CAR	*blaOXA-23*	WP_001046004.1 and others
	OXA-23 family carbapenem-hydrolyzing class D beta-lactamase OXA-239			*blaOXA-239*	WP_063862190.1 and others
	carbapenem-hydrolyzing class D beta-lactamase OXA-24		CAR	*blaOXA-24*	WP_012754353.1 and others
	OXA-24 family carbapenem-hydrolyzing class D beta-lactamase OXA-72			*blaOXA-72*	WP_000713530.1 and others
	OXA-51 family carbapenem-hydrolyzing class D beta-lactamase OXA-51		CAR	*blaOXA-51*	WP_002033109.1 and others
	OXA-51 family carbapenem-hydrolyzing class D beta-lactamase OXA-64			*blaOXA-64*	WP_001021788.1 and others
	OXA-51 family carbapenem-hydrolyzing class D beta-lactamase OXA-65			*blaOXA-65*	WP_001021782.1 and others
	OXA-51 family carbapenem-hydrolyzing class D beta-lactamase OXA-66			*blaOXA-66*	WP_001021792.1 and others
	OXA-51 family carbapenem-hydrolyzing class D beta-lactamase OXA-68			*blaOXA-68*	WP_001021775.1 and others
	OXA-51 family carbapenem-hydrolyzing class D beta-lactamase OXA-69			*blaOXA-69*	WP_001021779.1 and others
	OXA-51 family carbapenem-hydrolyzing class D beta-lactamase OXA-71			*blaOXA-71*	WP_001021785.1 and others
	OXA-51 family carbapenem-hydrolyzing class D beta-lactamase OXA-82			*blaOXA-82*	WP_001021793.1 and others
	OXA-51 family carbapenem-hydrolyzing class D beta-lactamase OXA-90			*blaOXA-90*	WP_001021781.1 and others
	OXA-51 family carbapenem-hydrolyzing class D beta-lactamase OXA-91			*blaOXA-91*	WP_001021776.1 and others
	OXA-51 family carbapenem-hydrolyzing class D beta-lactamase OXA-94			*blaOXA-94*	WP_029424390.1 and others
	OXA-51 family carbapenem-hydrolyzing class D beta-lactamase OXA-95			*blaOXA-95*	WP_031960432.1 and others
	OXA-51 family carbapenem-hydrolyzing class D beta-lactamase OXA-98			*blaOXA-98*	WP_001021777.1 and others
	OXA-51 family carbapenem-hydrolyzing class D beta-lactamase OXA-100			*blaOXA-100*	WP_001021795.1 and others
	OXA-51 family carbapenem-hydrolyzing class D beta-lactamase OXA-104			*blaOXA-104*	WP_024433915.1 and others
	OXA-51 family carbapenem-hydrolyzing class D beta-lactamase OXA-120			*blaOXA-120*	WP_004738885.1 and others
	OXA-51 family carbapenem-hydrolyzing class D beta-lactamase OXA-223			*blaOXA-223*	WP_001022758.1 and others
	OXA-51 family carbapenem-hydrolyzing class D beta-lactamase OXA-259			*blaOXA-259*	WP_001021784.1 and others
	OXA-51 family carbapenem-hydrolyzing class D beta-lactamase OXA-371			*blaOXA-371*	WP_063862738.1 and others
	OXA-51 family carbapenem-hydrolyzing class D beta-lactamase OXA-402			*blaOXA-402*	WP_001021789.1 and others
	carbapenem-hydrolyzing class D beta-lactamase OXA-58		CAR	*blaOXA-58*	WP_002002480.1 and others
	OXA-58 family carbapenem-hydrolyzing class D beta-lactamase OXA-96			*blaOXA-96*	WP_063864543.1
	OXA-134 family carbapenem-hydrolyzing class D beta-lactamase OXA-235		CAR	*blaOXA-235*	WP_000854009.1 and others
	OXA-134 family carbapenem-hydrolyzing class D beta-lactamase OXA-237			*blaOXA-237*	WP_000854010.1 and others
	carbapenem-hydrolyzing class D beta-lactamase OXA-143		CAR	*blaOXA-143*	WP_063861042.1
	OXA-143 family carbapenem-hydrolyzing class D beta-lactamase OXA-253			*blaOXA-253*	WP_032495764.1
Efflux pumps	multidrug efflux RND transporter AdeABC outer membrane channel subunit AdeC		CEPH, CAR	*adeC*	WP_000047249.1 and others
	*Acinetobacter baumannii* efflux resistant AdeR			*adeR_A91V*	WP_039198290.1 and others
	*Acinetobacter baumannii* efflux resistant AdeR			*adeR_P56S*	WP_088753133.1 and others
	*Acinetobacter baumannii* efflux resistant AdeR			*adeR_P116L*	WP_111853508.1 and others
	*Acinetobacter baumannii* efflux resistant AdeS			*adeS_G336S, adeS_N125K*	WP_031975145.1, WP_057691178.1 and others
	*Acinetobacter baumannii* efflux resistant AdeS			*adeS_H189Y*	WP_119491814.1, WP_000837466.1, WP_046882653.1
Penicillin-binding proteins	*Acinetobacter baumannii* carbapenem resistant FtsI		CAR	*ftsI_A515V*	WP_000227939.1 and others (penicillin-binding protein PBP3)

CEPH = cephalosporins; CAR = carbapenems.

**Table 2:** Mechanisms of *A. baumannii* resistance to aminoglycosides.

	Element Name and Symbol	Resistance	Gene	Protein Products
Aminoglycoside acetyltransferases	Aminoglycoside 2’-N-acetyltransferase AAC(2’)-Ib	GEN, TOB, DIB, NET [101]	*aac(2′)-Ib*	WP_001159732.1 ^†^
	Aminoglycoside 3-N-acetyltransferase	GEN	*aac(3)*	WP_195206917.1
	AAC(3)-I family aminoglycoside 3-N-acetyltransferase	GEN	*aac(3)-I*	HAV6561382.1, WP_069597335.1
	Aminoglycoside N-acetyltransferase AAC(3)-Ia	AST, GEN, SIS [102]	*aac(3)-Ia*	WP_002089484.1, and others
	Aminoglycoside N-acetyltransferase AAC(3)-IId	GEN	*aac(3)-IId*	WP_000557454.1, WP_126562472.1
	Aminoglycoside N-acetyltransferase AAC(3)-IIe	GEN	*aac(3)-IIe*	WP_000557452.1, WP_002063884.1, WP_033107705.1, WP_095530619.1, and others
	Aminoglycoside N-acetyltransferase AAC(3)-Iva	APR, GEN, TOB	*aac(3)-IVa*	WP_001199192.1
	Aminoglycoside 6’-N-acetyltransferase	all	*aac(6’)*	HAV4276337.1
	Aminoglycoside N-acetyltransferase AAC(6’)-31	all	*aac(6’)-31*	WP_044424439.1
	Aminoglycoside 6’-N-acetyltransferase AAC(6’)-33	all	*aac(6’)-33*	WP_015059044.1
	AAC(6’)-Ia family aminoglycoside 6’-N-acetyltransferase	AMI, KAN, TOB, putatively against all	*aac(6’)*	EGY2236091.1, EGY5968849.1, WP_088756823.1, WP_088774065.1, WP_140976846.1
	AAC(6’)-Ia family aminoglycoside 6’-N-acetyltransferase AacA16	all	*aacA16*	WP_001109644.1
	Aminoglycoside 6’-N-acetyltransferase AacA34	all	*aacA34*	WP_052285801.1
	AAC(6’)-Ia family aminoglycoside 6’-N-acetyltransferase AacA43	KAN, TOB	*aacA43*	WP_024437351.1
	Aminoglycoside N-acetyltransferase AAC(6’)-Ian	AMI, KAN, TOB, putatively against all	*aac(6’)-Ian or aacA57-2*	WP_000960976.1
	AAC(6’)-Ib family aminoglycoside 6’-N-acetyltransferase	AMI, DIB, GEN, ISE, KAN, NET, SIS, TOB [102]	*aac(6’)-Ib*	WP_063840280.1, and others
	Aminoglycoside N-acetyltransferase AAC(6’)-Ib’	GEN	*aac(6’)-Ib’*	WP_014454105.1
	AAC(6’)-Ighjkrstuvwx family aminoglycoside N-acetyltransferase	AMI, KAN, TOB	*aac(6’)-I*	WP_169109636.1, WP_150956588.1, WP_005288246.1, WP_005243483.1
	Aminoglycoside N-acetyltransferase AAC(6’)-Ib3	AMI, KAN, TOB	*aac(6’)-Ib3*	WP_032488579.1
	Aminoglycoside N-acetyltransferase AAC(6’)-Ib4	GEN	*aac(6’)-Ib4*	WP_003159191.1
	aminoglycoside N-acetyltransferase AAC(6’)-Ih	AMI, KAN, TOB	*aac(6’)-Ih*	WP_016541245.1 ^†^
	Aminoglycoside N-acetyltransferase AAC(6’)-Il	AMI, KAN, TOB	*aac(6’)-Il*	WP_156193962.1
	AAC(6’)-II family aminoglycoside 6’-N-acetyltransferase AacA35	GEN, KAN, TOB	*aacA35*	WP_024437054.1
	Aminoglycoside N-acetyltransferase AAC(6’)-IIc	GEN, KAN, TOB	*aac(6’)-IIc*	WP_149959345.1, WP_149938250.1
	Fluoroquinolone-acetylating aminoglycoside 6’-N-acetyltransferase AAC(6’)-Ib-cr	AMI, KAN, TOB, QUI	*aac(6’)-Ib-cr*	WP_185936887.1
	Fluoroquinolone-acetylating aminoglycoside 6’-N-acetyltransferase AAC(6’)-Ib-cr5	AMI, KAN, TOB, QUI	*aac(6’)-Ib-cr5*	WP_063840321.1
Aminoglycoside adenyltransferases	Aminoglycoside nucleotidyltransferase ANT(2’’)-Ia	DIB, GEN, KAN, SIS, TOB [102]	*ant(2’’)-Ia*	WP_000381802.1, and others
	ANT(3’’)-I family aminoglycoside nucleotidyltransferase	STR, SPE	*ant(3’’)*	WP_038350223.1
	ANT(3’’)-Ia family aminoglycoside nucleotidyltransferase AadA	STR, SPE	*ant(3’’)-Ia*	WP_001205725.1
	ANT(3’’)-Ia family aminoglycoside nucleotidyltransferase AadA1	STR	*aadA1*	WP_001206316.1, and others
	ANT(3’’)-Ia family aminoglycoside nucleotidyltransferase AadA2	STR	*aadA2*	WP_001206356.1, WP_001261740.1, and others
	ANT(3’’)-Ia family aminoglycoside nucleotidyltransferase AadA5	STR	*aadA5*	WP_000503573.1, WP_000503574.1
	ANT(3’’)-Ia family aminoglycoside nucleotidyltransferase AadA11	STR	*aadA11*	WP_048608579.1, HAV4466908.1
	ANT(3’’)-Ia family aminoglycoside nucleotidyltransferase AadA13	STR	*aadA13*	WP_001424636.1 ^†^
	ANT(3’’)-Ia family aminoglycoside nucleotidyltransferase AadA16	STR	*aadA16*	WP_001749984.1, WP_185936919.1
	ANT(3’’)-II family aminoglycoside nucleotidyltransferase	STR, SPE	*ant(3’’)-II*	WP_005240470.1
	Aminoglycoside nucleotidyltransferase ANT(3’’)-IIa	STR, SPE	*ant(3’’)-IIa*	WP_001279062.1, WP_001279061.1,WP_001112625.1, and others
	Aminoglycoside nucleotidyltransferase ANT(3’’)-IIc	STR, SPE	*ant(3’’)-IIc*	WP_005281276.1
Aminoglycoside phosphotransferases	APH(3’) family aminoglycoside O-phosphotransferase	all	*aph(3’)*	WP_196077463.1
	Aminoglycoside O-phosphotransferase APH(3’)-Ia	GEN, KAN, NEO, PAR, LIV, RIB [102]	*aph(3’)-Ia*	WP_000018326.1, and others
	APH(3’)-II family aminoglycoside O-phosphotransferase	KAN	*aph(3’)-II*	WP_000262467.1
	Aminoglycoside O-phosphotransferase APH(3’)-IIa	KAN	*aph(3’)-IIa*	WP_000572405.1, WP_171502934.1, and others
	APH(3’)-VI family aminoglycoside O-phosphotransferase	AMI, KAN	*aph(3’)-VI*	WP_014386410.1, and others
	Aminoglycoside O-phosphotransferase APH(3’)-VIa	AMI, KAN	*aph(3’)-VIa*	WP_000422636.1, and others
	Aminoglycoside O-phosphotransferase APH(3’)-VIb	AMI, KAN	*aph(3’)-VIb*	WP_000422633.1, WP_000422632.1, and others
	Aminoglycoside O-phosphotransferase APH(3’’)-Ib	STR	*aph(3’’)-Ib*	WP_001082319.1, and others
	Aminoglycoside O-phosphotransferase APH(4)-Ia	HYG	*aph(4)-Ia*	WP_185218783.1
	Aminoglycoside O-phosphotransferase APH(6)-Id	STR	*aph(6)-Id*	WP_000480968.1, and others
Target mutation: 16S rRNA methylase genes	ArmA family 16S rRNA (guanine(1405)-N(7))-methyltransferase	GEN	*armA*	WP_000359986.1, and others
	16S rRNA (guanine(1405)-N(7))-methyltransferase RmtB1	all	*rmtB and rmtB1*	WP_012372818.1
	RmtE family 16S rRNA (guanine(1405)-N(7))-methyltransferase	all	*rmtE*	WP_120494548.1
Efflux pump overactivity	Multidrug efflux MFS transporter AmvA	Putatively against all	*amvA*	WP_001170321.1, and others
	Multidrug efflux RND transporter AdeABC outer membrane channel subunit AdeC		*adeC*	WP_000047249.1, and others
	Multidrug efflux RND transporter periplasmic adaptor subunit AdeD		*adeD*	WP_002119008.1, WP_039254548.1
	Multidrug efflux RND transporter permease subunit AdeE		*adeE*	WP_002118518.1, WP_039254549.1
	Efflux system DNA-binding response regulator transcription factor AdeR		*adeR*	WP_032002707.1, EGY8404952.1,WP_020752724.1
	Two-component sensor histidine kinase AdeS two-component sensor histidine kinase		*adeS*	WP_031975145.1, and others
	Multidrug efflux SMR transporter EmrE		*emrE*	WP_109847152.1

AMI = amikacin; APR = apramycin; AST = astromicin; DIB = dibekacin; DNA = deoxyribonucleic acid; GEN = gentamicin; HYG = hygromycin; ISE = isepamicin; KAN = kanamycin; LIV = lividomycin; MFS = major facilitator superfamily; NEO = neomycin; NET = netilmicin; PAR = paromomycin; QUI = quinolone; RIB = ribostamycin; RNA = ribonucleic acid; RND = resistance/nodulation/cell division family; SIS = sisomicin; SMR = small multidrug resistance family; SPE = spectinomycin; STR = streptomycin; TOB = tobramycin; ^†^ = Identified via NCBI Identical Protein Groups, but with no respective mention in MicroBIGG-E.

**Table 3:** Tetracycline-specific mechanisms of *A. baumannii* resistance.

Element name and symbol	Resistance	Gene	Protein products
Tetracycline efflux MFS transporter Tet(39)	DOX, TET [110,111]	*tet(39)*	WP_004856455.1, and others
Tetracycline efflux MFS transporter Tet(A)	DOX, MIN, TET, TIG *	*tet(A)*	WP_000804064.1, and others
tetracycline efflux MFS transporter Tet(B)	DOX, MIN, TET [111]	*tet(B)*	WP_001089072.1, and others
Tetracycline efflux MFS transporter Tet(C)	TET [112]	*tet(C)*	WP_000841448.1
Tetracycline efflux MFS transporter Tet(D)	TET [113]	*tet(D)*	WP_024436252.1
Tetracycline efflux MFS transporter Tet(G)	DOX, MIN, TET	*tet(G)*	WP_001257840.1
Tetracycline efflux MFS transporter Tet(H)	OXY, TET [114]	*tet(H)*	WP_006248867.1
Tetracycline resistance ribosomal protection protein Tet(M)	TET, MIN	*tet(M)*	WP_000691727.1
Tetracycline-inactivating monooxygenase Tet(X)	all	*tet(X)*	WP_024160783.1, and others

DOX = doxycycline; MFS = major facilitator superfamily; MIN = minocycline; OXY = oxytetracycline; TET = tetracycline; TIG = tigecycline; * = only when RND-pumps AdeABC and AdeIJK are present.

**Table 4:** Quinolone-specific mechanisms of *A. baumannii* resistance.

Element Name and Symbol	Resistance	Gene	Protein Products
Fluoroquinolone-acetylating aminoglycoside 6’-N-acetyltransferase AAC(6’)-Ib-cr	CIP, NOR	*aac(6’)-Ib-cr*	WP_185936887.1
Fluoroquinolone-acetylating aminoglycoside 6’-N-acetyltransferase AAC(6’)-Ib-cr5	CIP, NOR	*aac(6’)-Ib-cr5*	WP_063840321.1
*A. baumannii* quinolone resistant GyrA (DNA gyrase subunit A)	CIP, LEV	*gyrA_S81L*	WP_000116444.1, and others
*A. baumannii* quinolone resistant ParC (DNA topoisomerase IV subunit A)	CIP, LEV	*parC_E88K*	WP_000202265.1, and others
*A. baumannii* quinolone resistant ParC (DNA topoisomerase IV subunit A)	CIP, LEV	*parC_S84F*	WP_000202250.1, and others
*A. baumannii* quinolone resistant ParC (DNA topoisomerase IV subunit A)	CIP, LEV	*parC_S84L*	WP_000202252.1, and others
QnrA family quinolone resistance pentapeptide repeat protein	CIP	*qnrA*	HAV5951840.1
QnrB family quinolone resistance pentapeptide repeat protein	CIP	*qnrB*	WP_185936934.1
Quinolone resistance pentapeptide repeat protein QnrB19	CIP	*qnrB19*	WP_012954666.1
QnrS family quinolone resistance pentapeptide repeat protein	CIP, *	*qnrS*	WP_147508156.1

CIP = ciprofloxacin; LEV = levofloxacin; NOR = norfloxacin; * = resistance to CIP, LEV, NOR, and enrofloxacin in the presence of rmtB [127].
